# The Montescano Effect: Being Resilient Through Challenges and Changes

**DOI:** 10.5964/ejop.8119

**Published:** 2022-02-25

**Authors:** Marina Maffoni, Paola Abelli, Giuseppe Laganga Senzio, Antonia Pierobon

**Affiliations:** 1 Psychology Unit of Montescano Institute, Istituti Clinici Scientifici Maugeri, IRCCS, Montescano (PV), Italy; 2 Health Management of Montescano Institute, Istituti Clinici Scientifici Maugeri, IRCCS, Montescano (PV), Italy; 3 Hospital Management of Pavia and Montescano Institutes, Istituti Clinici Scientifici Maugeri, IRCCS, Montescano (PV), Italy; Department of Psychology, Webster University, Geneva, Switzerland

**Keywords:** hospital system, interdisciplinary, humanistic approach, resilience, COVID-19

## Abstract

Hospitals are places where you live a piece of your life, no matter you are healthcare professional, patient or caregiver. This editorial describes the humanistic approach to medicine adopted by the Montescano Institute, an Italian research and clinical hospital dedicated to the rehabilitation of neurological and cardiopulmonary diseases according to updated international guidelines. The aim of these “notes from the field” is to provide a sound example of humanistic clinical practice before, during and after the challenges posed by the COVID-19 pandemic. In this environmental each individual is indeed engaged in relationships, which trigger mutual empowerment and growth.

“*Operations. Relations. Complications*”. This is the well-known tagline of a famous American medical drama (Quick, 2009) and this description of what happens in a hospital is not so distant from reality. Hospitals are places where nobody would stay as a patient and where professionals are requested to find solutions to medical puzzles and critical conditions, sometimes coming in terms with their own best expectations and intimate values. This is even more true in modern times, which is indelibly marked by the COVID-19 pandemic (Maffoni, Torlaschi, & Pierobon, 2020; Thornton, 2020). Hospitals are places where you live a piece of your life, no matter you are there for working or for recovering. There are much more than diseases and clinical investigations; there are human beings who are engaged in complicated relations with each other. For this reason, stories in each hospital are unique and unrepeatable, painful and hopeful at the same time. It is not easy to survive all of this. You must be resilient and ready to start again and again. You must be ready to transform yourself and to renew your body and spirit. If you can do all this, you probably arise refuelled with new perspectives and new opportunities. You can turn your own wounds into meaningful characteristics, taking the example of the old Japanese art of *kintsukuroi* in which broken objects are repaired with gold. Thus, scars are considered both unique elements giving sense to life and the starting point for a positive posttraumatic growth (Nijdam et al, 2018).

In this multifaceted scenario, providing humanistic care may play a crucial role for professionals and patients’ well-being. This approach to care has to be intended as the attempt to provide holistic care, going beyond the management of medical conditions only. In the 21st century, medicine should guarantee the best possible quality of life, allowing professionals and patients to find their own values, self-actualization and self-healing (Kogstad, Ekeland, & Hummelvoll, 2011; Shiau & Chen, 2008). On the one hand, professionals should be supported to find their own meaning of work and strategies to not be defeated by distress and malaise (Hynes et al., 2019; Maffoni, Argentero, et al., 2020; Maffoni, Zanatta et al., 2021). On the other hand, patients have to restore their body, but also their mind and spirit through sound relationships, attention to their existential needs and compassionate taking care (Kogstad et al., 2011; Grönblom-Lundström et al., 2019).

An example of humanistic care can be found in the North of Italy, where there is a little town of about four hundred inhabitants called Montescano which is surrounded by vineyards and rural traditions dominating daily life (Azzolini, 2021). Here, there is a well-known research and clinical hospital dedicated to the rehabilitation of neurological and cardiopulmonary diseases according to updated international guidelines and national health care system (Italian Ministry of Health, 2020). Briefly describing its history, this building was initially a mansion of a local noble family. Then, it constantly changed its clothes, rethinking its mission and its meaning. In the late 18th century, the hospital hosted and took care of children suffering from poliomyelitis. After Second World War until 1968, it was transformed into a sanatorium for children and adolescents developing tuberculosis. Moreover, after a brief period where the building was a retirement house, the building finally become one of the first Maugeri Institutes (Imbriani, 2005) which are hospitals providing multidisciplinary rehabilitation and occupational medicine to young and older adults. In 2020, the Montescano Institute and its inhabitants had to become a COVID-19 Unit, coping with the unpredictable challenges posed by the pandemic. Thus, the hospital changed its mission many times during the centuries.

Although this part of the story can be like many other hospitals, the atmosphere and characteristics of the Montescano Institute are something very rare. Actually, what makes the difference it is the beneficial effect of nature. Previous literature has already pinpointed that the connection with nature can both ignite positive emotions and support attention restoration, as well as stress reduction (Capaldi et al., 2015; Choe et al., 2020; Cleary et al., 2017; Martin et al., 2020). To this regard, one thing which goes right into the hearts of people is the country landscape surrounding everything and influencing the people lives. It is not so comfortable to reach the hospital as the street winds amongst fields and vineyards. There are no shops, nor amenities in the surroundings. However, on the summit of the hill, what you perceive is not poverty or deprivation rather unspoiled nature, sense of community and peace. Some patients decided to spend here even one or two months each year as a pleasant routine to manage their chronic conditions. It is not easy to describe in words what happens. Just think about a place where inpatients and outpatients are first involved in different medical assessments and programs of traditional and robotic multidisciplinary rehabilitation. Then, they can take a sunbathe between a coffee and a card game on the sunny terrace of the Institute or under the shadows of the magnolia trees of its park. During the weekend have been displayed some dancing afternoon for patients thanks to some professionals’ willingness to spend free time with patients. Similarly, at the end of a daily hard physiotherapy session, the therapist can turn on the music and dance with the older adult who was waiting for nothing but feeling young again. It can also happen that patients become friends of local farmers and buy their products and wine on their discharge. Furthermore, in the pre-COVID era, some hospitalised guests, without food restrictions, had a pizza delivered while watching the football match on TV or just to have a good time with other patients. Currently, due to COVID-19 restrictions, relatives cannot yet visit their hospitalized lovers within the unit. For this reason, it is a daily experience seeing families outside the hospital who talk and motivate the patients speaking through the window of their room. Moreover, during the epidemic peak outbreak, healthcare professionals proposed video calls and a psychological helpline to support patients and caregivers, trying to bridge the distance imposed by sanitary restrictions (Maffoni, Torlaschi et al., 2021).

Healthcare professionals working in this hospital are sort of a big family where there is mutual support, but also the time to squabble in case of different opinions. During the years, they challenged themselves by taking part in different research studies (Scientific IRCCS Network, 2021), as well as in activities such as mindfulness training experiences within the working place. Each day, professionals easily communicate with each other, exchanging patient’s updates or calling for a specialist consultation in front of a vending machine or just informally knocking on the colleague’s door. The Montescano atmosphere gradually captures even the most reticent professionals who are not used to this familiar approach. Thus, each professional knows the other colleagues and the majority of patients. It is like a big house with shared spaces and experiences, and this allows to put in practice the concept of interdisciplinarity through the biopsychosocial approach (Engel, 1980).

Overall, it can be said that Montescano provides what can be considered a therapeutic landscape, that is “the physical and built environments, social conditions and human perceptions combine to produce an atmosphere which is conducive to healing” (Gesler, 1996, p. 96, as cited in Cleary et al., 2017). Thus, the easy-going way to communicate and the familiar and human approach between people create a strong and unique alchemical poison improving the mood and, in turn, the clinical and rehabilitative outcomes. Professionals and patients are firstly human beings who are interrelated to each other by a common thread. The hospitalization takes place in a meditative place dominated by the nature which promotes the eudaimonic wellbeing of individual (Choe et al., 2020; Cleary et al., 2017). The entire building and its inhabitants are used to cope with adversities and unstoppable changes in medicine and society. It may be difficult, distressing and even painful, but, maybe, it is a winning way to survive and to improve the effects of therapies and subjective wellbeing (Pierobon et al., 2011).

In this vein, to address patient’s difficulties to reach the hospitals and increase the number of individuals taking in charge, future challenges for this hospital may be the daily adoption of telemedicine, tele-rehabilitation and high technology rehabilitation. Indeed, telemedicine will allow monitoring patients at their home. Similarly, tele-rehabilitation will enhance the benefit of rehabilitation, extending interventions from hospitals to home. Moreover, neuromotor rehabilitation using high technologies, robotics and virtual reality tools is described as promising. Specifically, high technology rehabilitation can actually maximise the benefit of standard rehabilitation programs and guarantee the patient’s social and personal needs (Voinescu, Sui, & Stanton Fraser, 2021).

Besides these future perspectives, the healthcare approach adopted by this Institute can provide useful insights in this tough time. Unfortunately, the COVID-19 pandemic teaches us to see hospitals as places of grief and death. However, it has not to be so. Hospitals can be considered new starting points characterised by smiles, hope, new awareness and challenges. The healthcare landscape need not only for medicine but also for humanistic approaches to medicine (Ferry-Danini, 2018). Moreover, promoting and discovering the mechanisms within the connection between nature and subjective wellbeing can be crucial to promote more targeted solutions for patients and to maximise the clinical outcome and psychophysical wellbeing (Capaldi et al., 2015; Cleary et al., 2017; Martin et al., 2020).

Thus, when a new patient or a professional come here for the first time, they usually say: “It doesn’t seem to be a common hospital, is it?!”. Veterans just respond saying “Oh yeah, we know, it’s the atmosphere of this hospital which, maybe, will spread all around one day … It is the Montescano Effect!”.
